# N6-Methyladenosine-Related lncRNAs Are Novel Prognostic Markers and Predict the Immune Landscape in Acute Myeloid Leukemia

**DOI:** 10.3389/fgene.2022.804614

**Published:** 2022-05-09

**Authors:** Lulu Zhang, Wen Ke, Pin Hu, Zhangzhi Li, Wei Geng, Yigang Guo, Bin Song, Hua Jiang, Xia Zhang, Chucheng Wan

**Affiliations:** ^1^ Department of Hematology, Taihe Hospital, Hubei University of Medicine, Shiyan, China; ^2^ Department of Osteology, Taihe Hospital, Hubei University of Medicine, Shiyan, China

**Keywords:** m6A modification, long non-coding RNA, acute myeloid leukemia, prognosis, immune infiltration, copy number variation

## Abstract

**Background:** Acute myelocytic leukemia (AML) is one of the hematopoietic cancers with an unfavorable prognosis. However, the prognostic value of N 6-methyladenosine-associated long non-coding RNAs (lncRNAs) in AML remains elusive.

**Materials and Methods:** The transcriptomic data of m6A-related lncRNAs were collected from The Cancer Genome Atlas (TCGA) and the Gene Expression Omnibus (GEO) database. AML samples were classified into various subgroups according to the expression of m6A-related lncRNAs. The differences in terms of biological function, tumor immune microenvironment, copy number variation (CNV), and drug sensitivity in AML between distinct subgroups were investigated. Moreover, an m6A-related lncRNA prognostic model was established to evaluate the prognosis of AML patients.

**Results:** Nine prognosis-related m6A-associated lncRNAs were selected to construct a prognosis model. The accuracy of the model was further determined by the Kaplan–Meier analysis and time-dependent receiver operating characteristic (ROC) curve. Then, AML samples were classified into high- and low-risk groups according to the median value of risk scores. Gene set enrichment analysis (GSEA) demonstrated that samples with higher risks were featured with aberrant immune-related biological processes and signaling pathways. Notably, the high-risk group was significantly correlated with an increased ImmuneScore and StromalScore, and distinct immune cell infiltration. In addition, we discovered that the high-risk group harbored higher IC50 values of multiple chemotherapeutics and small-molecule anticancer drugs, especially TW.37 and MG.132. In addition, a nomogram was depicted to assess the overall survival (OS) of AML patients. The model based on the median value of risk scores revealed reliable accuracy in predicting the prognosis and survival status.

**Conclusion:** The present research has originated a prognostic risk model for AML according to the expression of prognostic m6A-related lncRNAs. Notably, the signature might also serve as a novel biomarker that could guide clinical applications, for example, selecting AML patients who could benefit from immunotherapy.

## Introduction

Acute myelocytic leukemia (AML) is one of the most common hematopoietic malignancies in adults. AML is featured with the infiltration of malignant myeloid progenitor cells into the peripheral blood, bone marrow, and other tissues, which often progress rapidly leading to poor prognosis (De Kouchkovsky and Abdul-Hay, 2016; Papaemmanuil et al., 2016). In the past 15 years, basic and translational research, such as large-scale genomic analyses, has greatly improved our understanding of the molecular and genetic pathogenesis of AML. As a result, many new and effective targeted therapies have been developed. These include protein kinase small-molecule inhibitors such as gilteritinib (Perl et al., 2019), enasidenib (Stein et al., 2017), and ivosidenib (Dinardo et al., 2018); immune checkpoint antibody such as gemtuzumab (Vago and Gojo, 2020); and mitochondrial inhibitor venetoclax (Candon, 2018; Ram et al., 2019). Despite these advances, the prognosis of AML is still poor with a 5-year overall survival (OS) rate of approximately 10% (Dewolf and Tallman, 2020). Therefore, there is a great need to learn more insights into the molecular pathogenesis of AML for the development of novel therapies.

N 6-Methyladenosine (m6A) RNA modification has been reported to play an important role in AML *via* multiple pathophysiological mechanisms (Zheng and Gong, 2021). Numerous studies have described that methyltransferases (“writers”) [METTL3 (Vu et al., 2017), METTL14 (Weng et al., 2018), WTAP (Naren et al., 2021)], demethylases (“erasers”) [FTO (Qing et al., 2021a), ALKBH5 (Shen et al., 2020; Wang et al., 2020)], and the common m6A binding protein (“reader”) [YTHDF2 (Paris et al., 2019)] were significantly upregulated in different subtypes of AML. These findings indicated that the transcriptome level of m6A regulators may be important diagnostic and prognostic biomarkers for AML patients. Moreover, the overexpression of IGF2BP1 (“reader”) promoted the chemotherapeutic resistance of tumor cells by activating many stemness-related signaling *via* transcription regulation or metabolic reprogramming (Elcheva et al., 2020). Interestingly, the inhibition of METTL14, FTO, ALKBH,5 or YTHDF2 only weakly interrupted normal hematopoiesis compared with leukemogenesis. In addition, m6A was reported to be crucial in the initiation and progression of AML, indicating that targeting m6A regulators could be used for the elimination of leukemia cells. It has been reported that inhibiting FTO could decrease the expression level of immune checkpoints in an m6A-dependent manner, which significantly increased the AML cell sensitivity to T-cell-based killing and overcoming decitabine-mediated immune evasion (Su et al., 2020). Nonetheless, the underlying mechanisms by which m6A regulate AML pathophysiology remains to be clarified in order to develop effective and safe pharmaceutical inhibitors targeting m6A regulators.

There were several long non-coding RNAs (lncRNAs) that have been implicated with the growth, metastasis, and apoptotic cell death of AML cells, thus affecting the progression of AML and patient’s survival. For example, linc00239 has been shown to downregulate the efficacy of doxorubicin and negatively regulate the apoptosis of AML cells (Yang et al., 2019). lncRNA HOTTIP (Zhuang et al., 2019) was specifically upregulated in the microenvironment of bone marrow in patients with the AML M5 subtype. Furthermore, some lncRNAs were considered as tumor-resistant factors in AML. For example, lncRNA H22954 was decreased in the bone marrow of AML patients, which was correlated with an increased risk of disease relapse. H22954 upregulation blocked AML proliferation and promoted cell apoptosis. Furthermore, H22954 overexpression decreased tumor growth in animal models *via* the BCL-2-dependent mechanism (Qi et al., 2019). Nonetheless, the role of m6A-associated lncRNAs in AML remains unclear. Thus, understanding the expression pattern and prognostic role of m6A-associated lncRNAs in AML is of great importance.

In the present study, we established an m6A-related lncRNA-based prognostic model (FAM30A, HCP5, LINC00342, LINC00963, MEG3, HCG18, TMEM147-AS1, N4BP2L2-IT2, and TTTY15) which harbored a satisfied accuracy in predicting the OS of patients with AML in an independent manner. We also divided the AML patients into two clusters based on the risk scores of the model. Gene set enrichment analysis (GSEA) indicated that increased risk scores were associated with immune-related biological processes and signaling pathways. Our results also demonstrated a higher risk score was highly related to increased ImmuneScore and StromalScore, and distinct immune cell infiltration. The analysis of copy number data showed that the low-risk group was mainly manifested with gene amplification; however, the high-risk group was more associated with the censoring of gene copy numbers. Notably, the samples with increased risk scores harbored higher IC50 values of multiple chemotherapeutic drugs.

## Materials and Methods

### Data Preparation and Preprocessing

Transcriptome materials in fragments per kilobase of transcript per million mapped reads (FPKM) form of AML was collected from The Cancer Genome Atlas (TCGA) GDC data portal (https://portal.gdc.cancer.gov/). Then, transcripts were labeled with either mRNA or lncRNA and extracted as an independent matrix, respectively. Furthermore, the FPKM value was converted to transcripts per million read (TPM) value. Clinical information, including age, gender, and survival of the corresponding patients, was acquired from TCGA. After the deletion of samples with incomplete clinical information, 130 tumor tissues were included in the present study. In addition, the copy number variations (CNVs) of these patients were downloaded in masked somatic mutation annotation, which was utilized to visualize somatic mutations using R package “maftools” (Mayakonda et al., 2018).

Microarray data GSE37642 (Li et al., 2013; Herold et al., 2014; Kuett et al., 2015; Herold et al., 2018) was extracted from the Gene Expression Omnibus (GEO) database (http://www.ncbi.nlm.nih.gov/geo). GSE37642 was retrieved from the GPL570 platform (HG-U133_Plus_2) Affymetrix Human Genome U133 Plus 2.0 Array and GPL96 platform (HG-U133A) Affymetrix Human Genome U133A Array. The data type was microarray data, and the species was *Homo sapiens*, which included 562 tissue samples of AML (Supplementary Table S1). After the deletion of samples with incomplete survival information, 553 AML tissue samples were obtained. Subsequently, the mRNA and lncRNA expression data from TCGA and GEO were merged separately, and R package “sva” was applied for standardization and removing batch effects.

### M6A-Related lncRNA Risk Model Construction

The expression and co-expression of m6A-related genes in AML were studied. Next, we calculated the correlation coefficient and *p* value between m6A-related genes and lncRNA profiles, and m6A-related lncRNAs were identified according to the standard of *p* < 0.05 in the correlation analysis as mentioned in previous studies (Zhang P. et al., 2021; Zhao et al., 2021). Moreover, we incorporated the expression of m6A-related lncRNA in TCGA and the GEO database into model construction. R package “random Forest” was utilized to build a random forest model, which was an m6A-related lncRNA risk assessment model for AML patients, and divided AML patients into high- and low-risk groups according to the median of risk scores.

### Differentially Expressed Gene Analysis Based on Related Risk Scoring Model of m6A-Related lncRNA Genes

The DESeq2 algorithm (Love et al., 2014) and limma package (Ritchie et al., 2015) in Bioconductor were conducted for sequencing and microarray matrix to screen the DEGs between high- and low-risk groups of AML patients. The cutoff value for screening was |log fold change (FC)| > 0.5 and adjusted *p* value <0.05. Then, the selected DEGs were used to draw a heatmap and volcano plot. The Venn diagram displayed overlapped DEGs between two datasets.

### Functional and Pathway Enrichment Analysis

Gene ontology (GO) analysis serves as a bioinformatics tool that provides systematic annotations, containing molecular functions (MFs), biological processes (BPs), and cellular components (CCs). Kyoto encyclopedia of genes and genomes (KEGG) is a widely utilized database for understanding information about biological pathways, genomes, drugs, and diseases. GO annotations and KEGG pathway enrichment analysis of overlapped DEGs were conducted using the R package “clusterProfiler” (Yu et al., 2012), and items with a false discovery rate (FDR) < 0.05 were considered statistically significant.

In order to explore the differences in biological processes between different groups, we performed gene set enrichment analysis (GSEA) according to the gene expression profiles of AML samples. GSEA is a statistical algorithm to evaluate whether *a priori*-defined gene sets showed statistically significant and consistent differences between two different biological statuses (Subramanian et al., 2005). It is usually performed to estimate variations in pathway and biological process activity samples undergoing high-through sequencing. GSEA was performed based on the “h.all.v7.2.symbols.gmt” gene set (Subramanian et al., 2005) downloaded using the MSigDB database. Adjusted *p* value < 0.05 was considered as statistically significant.

### Identification of Intra-Tumoral Infiltrated Immune Cells and Correlation Analysis

The CIBERSORT algorithm and LM22 matrix (Newman et al., 2015) were applied to assess the percentage of immune–stromal components in the tumor microenvironment (TME) for each AML sample. A total of 22 distinct immune cell types in the TME of AML samples were distinguished as highly sensitive and specific. CIBERSORT is a deconvolution algorithm using transcriptomic data (with 547 characteristic genes) (Newman et al., 2015), which was considered to be the smallest representative of each cell type. We calculated the cellular components of tumor samples according to the gene expression profile of the tissue using CIBERSORT.

Meanwhile, the R package “ESTIMATE” (Yoshihara et al., 2013) was implemented to assess the immune activity in tumors. ESTIMATE analysis quantified the infiltrated immune cells in the tumor tissues according to their gene expression values and obtained the immune score for each sample. The differences in the features of immune infiltration in AML patients between the high- and low-risk groups were compared.

### Copy Number Variation Analysis

In order to analyze the copy number changes in groups with different risk scores of TCGA-AML patients, masked copy number segment data were downloaded using TCGAbiolinks package. GISTIC 2.0 was applied to perform analysis with downloaded CNV fragments through GenePattern5. During the analysis, except for a few parameters (e.g., the confidence setting is 0.99; the X chromosome is not excluded prior to the analysis), GISTIC 2.0 analysis chose the default settings. In the end, the Maftools package (Mayakonda et al., 2018) was conducted to visualize the analysis results of GISTIC 2.0.

### Analysis of Drug Sensitivity in Cancer

The Genomics of Drug Sensitivity in Cancer (GDSC) database (https://www.cancerrxgene.org/) (Yang et al., 2013) is a public database for cancer molecular therapy and mutation exploration. The cell line gene mutation data and IC50 values of different anticancer drugs were downloaded using R package “pRRophetic” (Geeleher et al., 2014), and then a ridge regression model was constructed to perform a correlation between patients with different risk scores and the sensitivity to different anticancer drugs.

### Construction of Clinical Prognostic Model Based on m6A-Related lncRNA Risk Model

In order to investigate whether the risk scores could reflect patients’ prognoses and serve as independent factors beyond clinicopathological characteristics, univariate and multivariate Cox regression analyses were thereafter conducted. A nomogram was depicted with risk scores and clinicopathological features incorporated. Harrell’s consistency index (C-index) was generated to quantify the discrimination performance. Subsequently, a calibration curve was generated to test the performance of the constructed nomogram by comparing the difference between the predicted and real OS of patients with AML. Moreover, decision curve analysis (DCA) was employed to evaluate the clinical values of the model.

### Statistical Analysis

Statistical analyses were conducted *via* R software (version4.0.2, http://www.R-project.org). When comparing two groups of continuous variables, Student’s t-test was utilized to study variables with normal distribution; however, variables without normal distribution were addressed using the Wilcoxon test. The chi-square test or Fisher’s exact test was applied to analyze the statistical difference between two groups of categorical variables. The correlation coefficient was calculated between different genes through Pearson correlation analysis. The Kaplan–Meier survival curves were depicted by R package “survival” and the differences of OS between different cohorts were assessed using the log-rank test. Univariate and multivariate Cox analyses were conducted to determine the independent factors for prognosis. *p* < 0.05 was considered statistically significant.

## Results

### Data Preprocessing

In order to explore the influence of m6A-related lncRNA on the occurrence and development of AML, we downloaded the transcriptome matrix of AML samples from TCGA and the GSE37642 dataset from the GEO database. Considering that the GSE37642 dataset contains two different annotation platforms, we organized the data of the two platforms and standardized for the gene expression (Supplementary Figures S1A–D). We further merged the transcriptome information from two platforms and removed batch effects (Supplementary Figures S1E, F).

### Construction of Risk Model According to the Expression Pattern of m6A-Related lncRNAs

**FIGURE 1:**
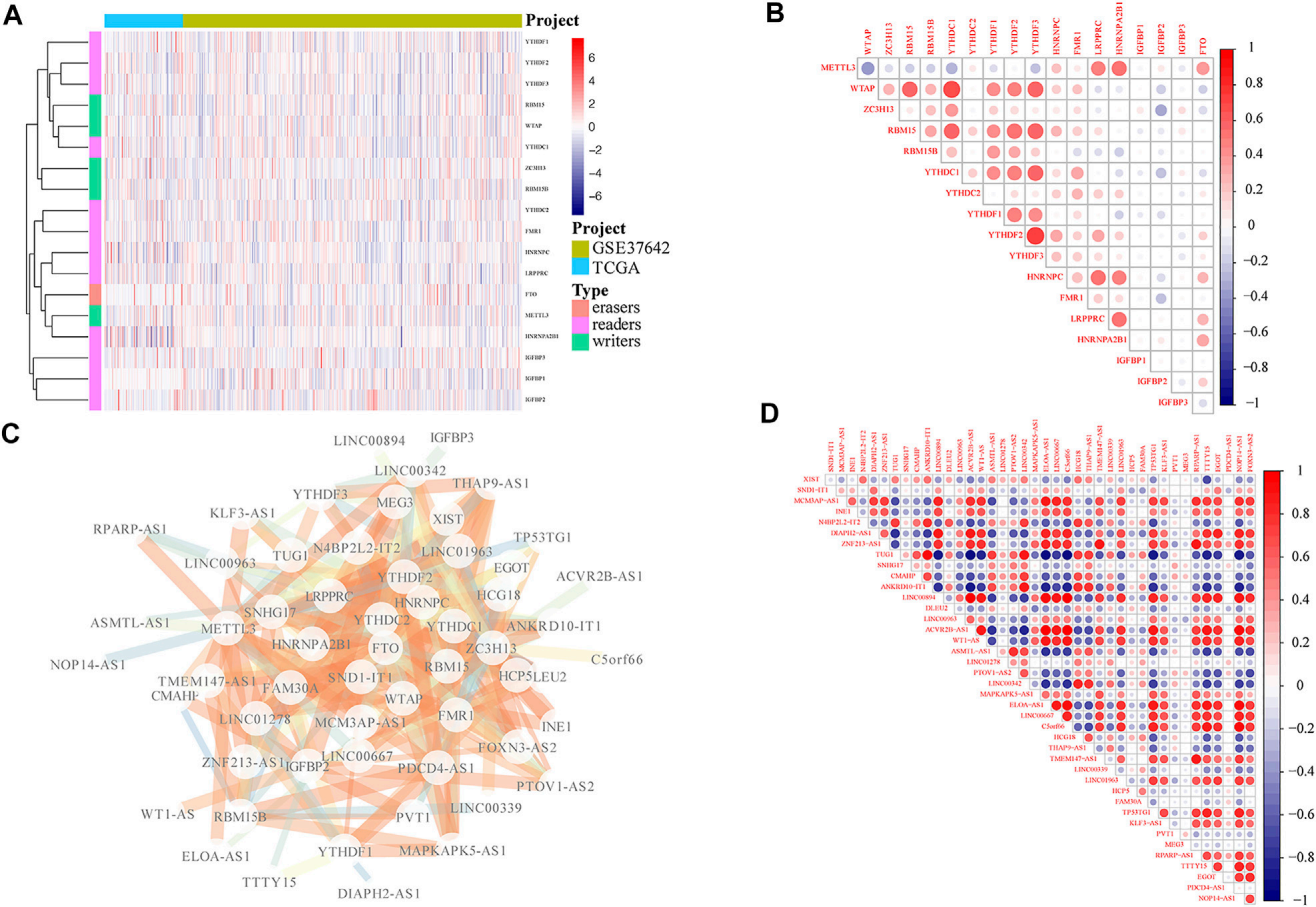
Screening of m6A-related lncRNAs. **(A)** Expression of m6A-related genes in AML patients from TCGA and GEO databases. **(B)** Heatmap showing the co-expression correlation of m6A-related gene in AML patients. **(C)** Based on correlation screening, 41 significant m6A-related lncRNAs were obtained. **(D)** Correlation analysis of the expression level of 41 significant m6A-related lncRNAs in AML patients in TCGA and GEO datasets, in which the color intensity was proportional to the degree of correlation; the deeper red color indicates more positive correlation, and the deeper blue color represents more negative correlation.

To investigate the expression pattern of m6A-related lncRNAs in AML, we first performed the comprehensive analysis according to the expression level of m6A-related genes in TCGA and GEO database and removed the batch effect. Heatmap and correlation analysis demonstrated the expression pattern of different m6A-related genes in AML (Figure 1A), even if the expressions of different m6A-related genes were mainly positively correlated, there was a negative association between METTL3 and WTAP with statistical significance (Figure 1B). According to the correlation analysis, we further searched for m6A-related lncRNA. A number of 41 m6A-related lncRNAs were finally obtained, and *p* < 0.05 was considered as significantly correlated genes (Figure 1C). Moreover, we conducted correlation analysis for m6A-related lncRNAs (Figure 1D).

**FIGURE 2:**
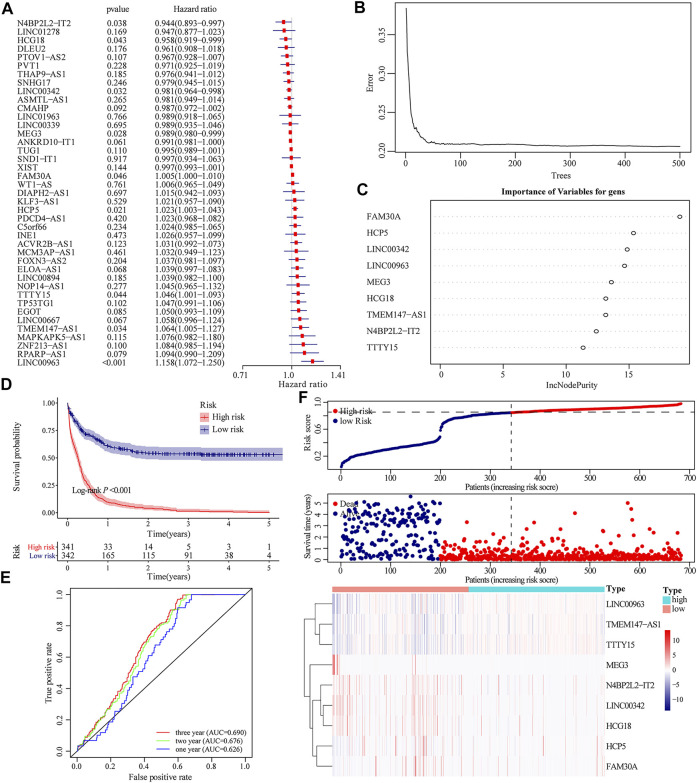
Construction of m6A-related lncRNA-based prognosis model. **(A)**Univariate Cox regression demonstrating that the expression level of nine lncRNAs were dramatically associated with the prognosis of AML patients. **(B)** Generating a random forest model according to the expression of m6A-related lncRNAs. **(C)** Weights of nine different m6A-related lncRNAs in the random forest model. **(D)** Kaplan–Meier curve assessing the impact of risk score on the OS rate of AML patients in TCGA and GEO database. **(E)** Time-dependent ROC curve evaluating the accuracy of the prognostic model. **(F)** Risk score distribution of AML patients in TCGA and GEO databases, and the heatmap shows the patient’s survival status and expression of characteristic genes.

We integrated the expression level of 41 m6A-related lncRNAs and implemented univariate Cox regression, of which 9 lncRNAs were prognosis-related, given the *p*-value was less than 0.05. Among them, the expressions of four lncRNAs were protective factors for the prognosis of patients, while other five lncRNAs were unfavorable factors (Figure 2A). According to the expression level of nine prognostic-related m6A lncRNAs, a risk scoring system was constructed by the random forest model. The risk score of the m6A-related lncRNAs that can quantitatively evaluate the impact on the prognosis of each AML patient was obtained (Figures 2B,C). Dividing all AML patients in TCGA dataset and GEO dataset into the high- and low-risk groups based on the cutoff value, which was the median of the risk scores for all samples. The K–M curve showed that compared to patients with higher risk scores, low-risk patients displayed a better prognosis (Log-rank *p* < 0.001, Figure 2D). The time-dependent receiver operating characteristic (ROC) curve further proved the accuracy of the prognostic model. The area under the curve (AUC) at 1-year, 2-year, and 3-year OS was 0.626, 0.676, and 0.690, respectively (Figure 2E). The distribution of risk scores, survival status, and the expression pattern of characteristic genes are presented in Figure 2F.

### Differentially Expressed Genes and Functional Enrichment Analysis Between High-Risk and Low-Risk Acute Myelocytic Leukemia

**FIGURE 3:**
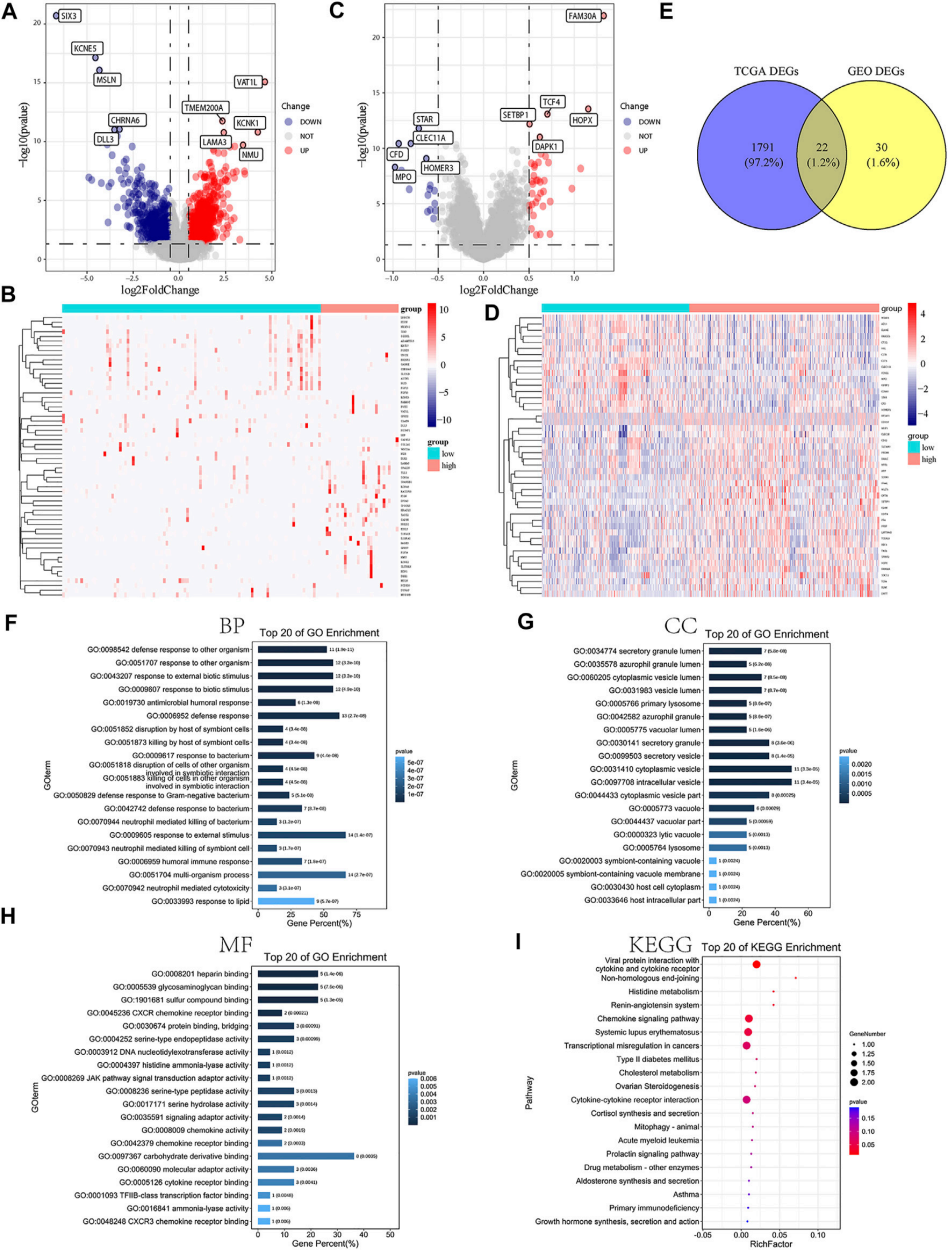
DEG analysis and functional enrichment analysis based on the related risk score model of m6A-related lncRNAs. **(A,B)** Volcano map and heatmap presenting the differential expression of DEGs between high- and low-risk AML patients in TCGA-AML dataset. **(C,D)** Volcano map and heatmap demonstrating the expression level of DEGs between high- and low-risk AML patients in the GEO dataset. **(E)** Venn diagram exhibiting 22 overlapped DEGs between TCGA-AML and GEO datasets. **(F)** GO BP terms suggesting the top 20 enriched biological processes. **(G)** GO CC terms demonstrating the top 20 enriched cellular components. **(H)** GO MF terms indicating the top 20 enriched molecular functions. **(I)** KEGG enrichment analysis showing the top 20 enriched pathways.

In order to illuminate the role of m6A-related lncRNA risk model on occurrence and progress of AML patients, AML samples included in the present study were divided into high- and low-risk groups based on the median of risk scores. We conducted differential analysis for the samples in two groups with the cutoff value set as log fold change |(FC)| > 0.5 and adjusted *p*-value <0.05. A total of 1,162 genes were significantly upregulated, while 651 genes were otherwise downregulated (Figures 3A,B) in the high-risk group of TCGA-AML cohort. In addition, 36 genes were definitely upregulated, while 16 genes, on the contrary, were dramatically downregulated (Figures 3C,D) in the high-risk group of the GEO cohort. Moreover, the intersection analysis displayed by the Venn plot showed 22 DEGs between two datasets (Figure 3E).

Subsequently, we conducted the functional enrichment analysis for the identified 22 DEGs. GO analysis showed that the significant DEGs were related to biological processes such as response to the external biotic stimulus, defense response, and humoral immune response (Figure 3F-H, Supplementary Table S2). KEGG analysis suggested that DEGs were mainly associated with the viral protein interaction with cytokine and cytokine receptors, chemokine signaling pathway, transcriptional misregulation in cancers, cytokine–cytokine receptor interaction pathways, and so on (Figure 3I, Supplementary Table S3). Furthermore, according to the differential analysis of TCGA cohort, the GSEA results indicated that myc targets, allograft rejection, apical junction, hypoxia, and PI3K/Akt/mTOR signaling pathway were significantly enriched in high-risk AML samples. The enrichment analysis of related pathways was displayed in Supplementary Figures S2A–I, Supplementary Table S4. All these demonstrated that increased risk scores were closely related to the cancer hallmark, malignancy, and immune pathways.

### Immune Cell Differentially Infiltrated in Samples With High- and Low-Risk Acute Myelocytic Leukemia Samples

**FIGURE 4:**
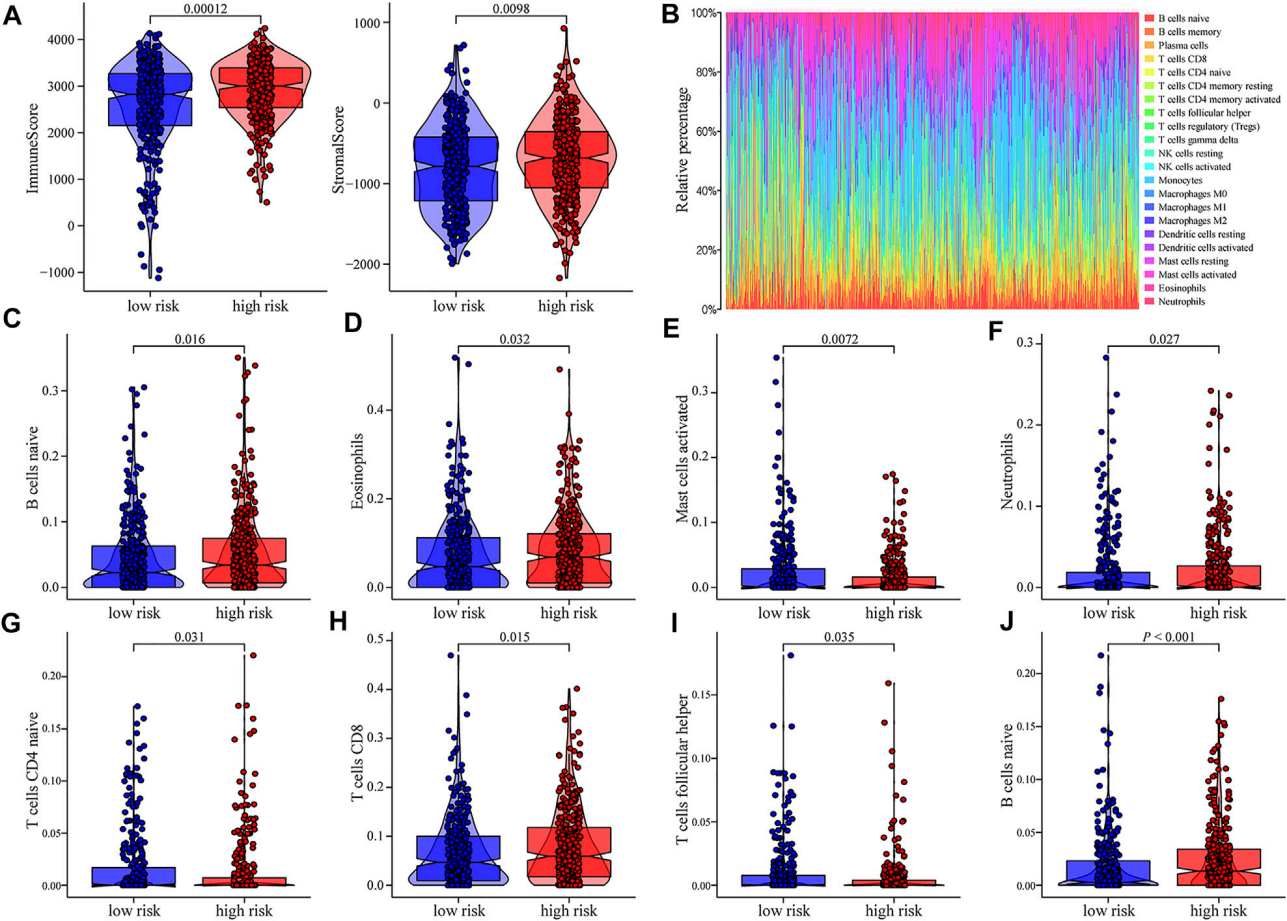
Relationship between risk scores and distinct immune cell infiltration. **(A)** Scatter diagram indicating the immune-related scores, and matrix-related scores of high-risk patient group were significantly increased (*p* < 0.05). **(B)** CIBERSORT was conducted to show the level of overall immune cell infiltration of AML patients in TCGA and GEO database. **(C–J)** Correlation analyses indicating that there were obvious differences in the expression of multiple immune cell subtypes between high- and low-risk groups of AML patients.

We investigated the impact of the m6A-related lncRNA risk model on the overall immune characteristics and immune cell infiltration in AML samples with different risk scores. Compared with the low-risk group, high-risk samples demonstrated higher ImmuneScore and StromalScore (ImmuneScore: *p* = 0.00012, StromalScore: *p* = 0.0098; Figure 4A). We further implemented the CIBERSORT algorithm to evaluate the infiltration level of 22 distinct immune cells (Figure 4B). Differential infiltrations were found in multiple immune cell subgroups between high- and low-risk groups, including naive B cells, eosinophils, activated mast cells, neutrophils, naive CD4 T cells, CD8 T cells, follicular helper T cells, and Tregs (Figure 4C–J).

### The Influence of Risk Score on the Genomic Changes of Acute Myelocytic Leukemia Patients

**FIGURE 5:**
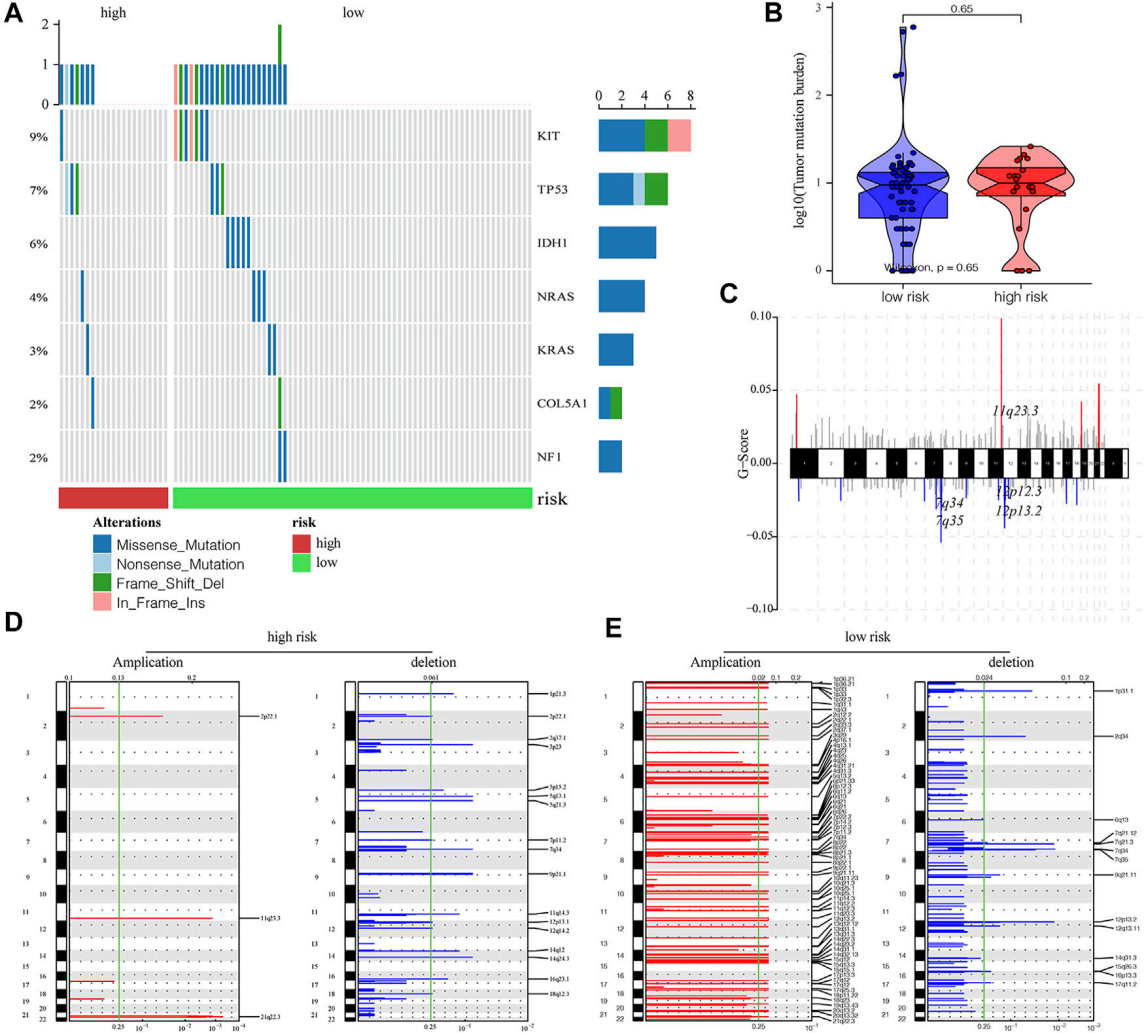
Influence of different m6A-related lncRNAs risk groups on the genetic variation of AML patients. **(A)** Mutation maps of common cancer driver genes between high- and low-risk groups. The waterfall chart displays the mutation information of every gene in each sample, of which various colors indicated different mutation types. The subsection before the legend demonstrated mutational burden. **(B)** Compared with the low-risk group, the high-risk group obtained a higher tumor mutation level, but the difference was not statistically significant (*p* = 0.65). **(C)** Overall level of CNV in TCGA-LAML patients. **(D,E)** Changes in the copy number levels of different genes between the high- and low-risk groups, among which red color represents the genes with significantly increased copy number, and blue color represents genes with significantly missing copy number.

Next, we evaluated the impact of m6A-related lncRNA risk score on the level of genetic variation, consisting of single-nucleotide polymorphism (SNP) and CNV among AML patients. The SNP analysis of driver genes in the occurrence of common tumors demonstrated that the SNP level of different driver genes was different between high- and low-risk groups (Figure 5A). Although patients with a higher risk had a relatively high tumor mutation burden (TMB), no significant differences were found between the two groups (*p* = 0.65; Figure 5B). We further discovered the overall CNV change level of TCGA-AML patients (Figure 5C). The results of CNV changing frequency suggested that patients with increased risk scores were mainly concentrated in the censoring of gene copy numbers (Figure 5D), while patients in the low-risk group were majorly manifested as gene amplification.

### Sensitivity of Acute Myelocytic Leukemia Patients to Different Small-Molecule Drugs Assessed by Risk Score Model

**FIGURE 6:**
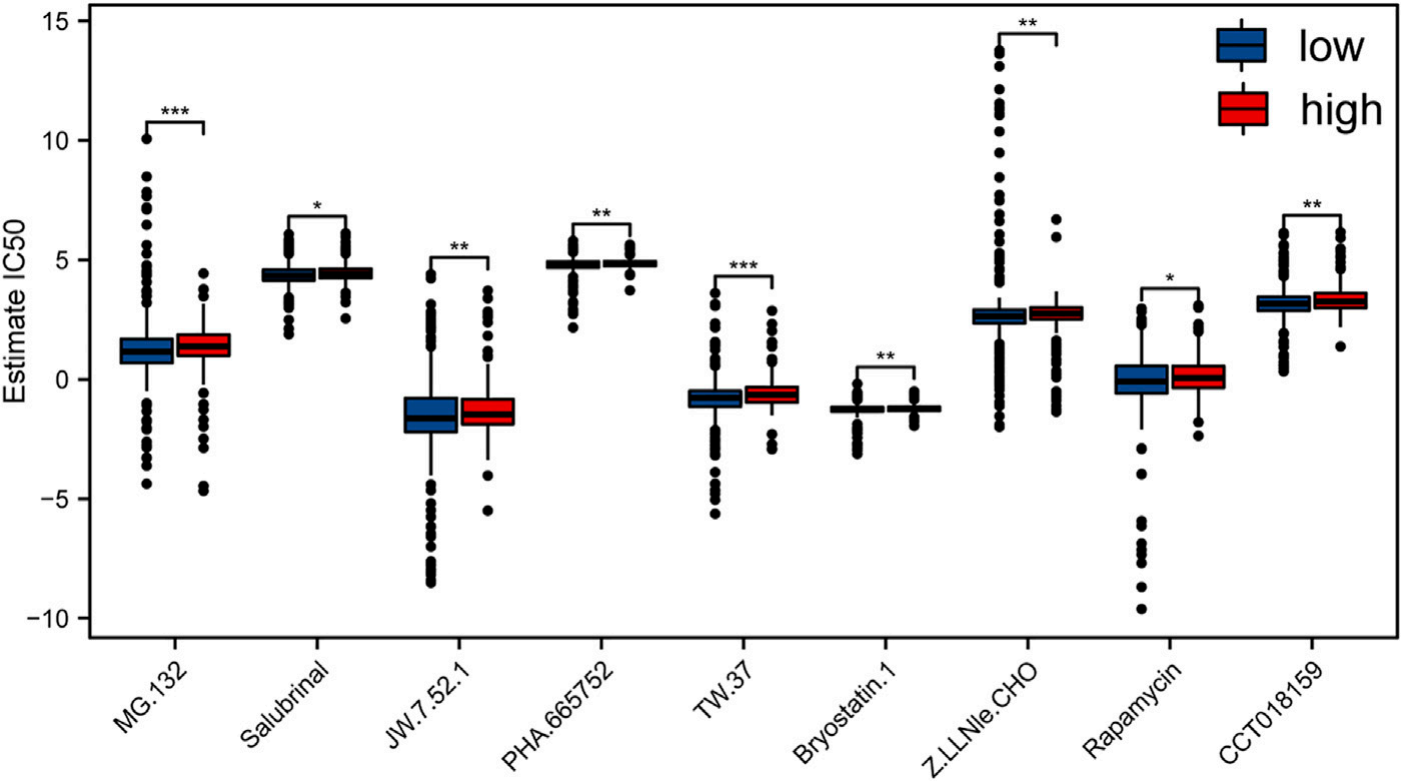
Evaluation of the sensitivity to different chemotherapeutics and small-molecule anticancer drugs with risk score of m6A-related lncRNAs according to the GDSC database.

In order to explore the sensitivity of patients with different risk scores to different drugs and small molecules, we downloaded the cell line gene mutation data and the IC50 values of different anticancer drugs from the GDSC database. The IC50 values of AML patients to different drugs were predicted based on the responsiveness of cell lines to 138 different chemotherapeutic drugs and small-molecule anticancer drugs. Taken together, our data indicated that the IC50 values of multiple chemotherapeutics and small-molecule anticancer drugs were obviously distinct between high- and low-risk samples (*p* < 0.01; Figure 6), especially TW.37 and MG.132.

### Construction of Clinical Prognostic Model Based on m6A-Related lncRNA Risk Score

**FIGURE 7:**
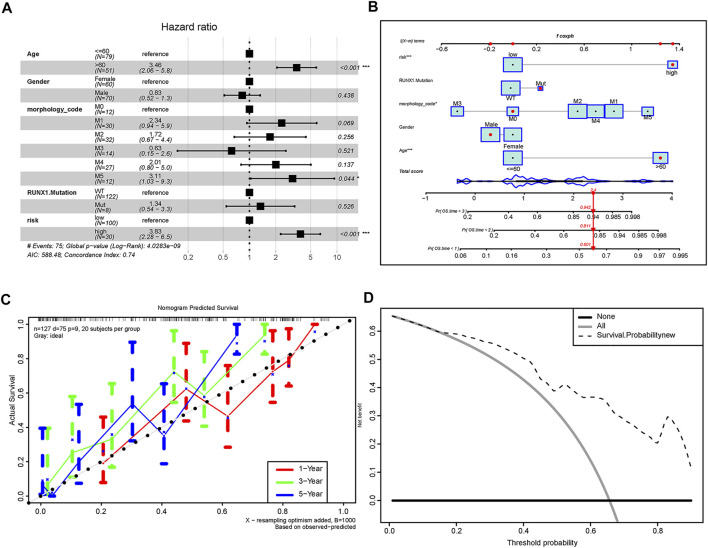
Predictive ability of the constructed model for the OS of AML patients. **(A)** Multivariate Cox regression analysis of risk scores combined with clinicopathological characteristics, which indicate that risk score, age, and M5 subgroup are independent risk factors for the prognosis of AML patients. **(B)** Nomogram showing clinicopathological characteristics such as age, gender, morphological subtype, RUNX1 mutation, and risk scores and manifesting great predictive accuracy for 1-, 2-, and 3-year survival rates. **(C)** Calibration curve of nomogram. X-axis indicates the survival situation predicted by the nomogram, and Y-axis indicates actual observed survival. Repeated 1,000 times in each run, the curve demonstrate that the model had an excellent predictive value for the prognosis of patients at 1, 3, and 5 years. **(D)** Decision curve analysis of nomogram; Y-axis indicates the net benefit, the dotted line indicates the benefit predicted by the model, the gray curve represents the benefit rate of all patients receiving the intervention, and the horizontal line indicates the benefit rate of the whole patients not received intervention. Take the intersection of the dotted line and all as the starting point, and the intersection with none as the end. Patients within this range can benefit from intervention.

Subsequently, we assessed the impact of the risk score on the OS of AML patients. Univariate and multivariate Cox regression analyses demonstrated that m6A-related lncRNA risk scores, age, and M5 subgroup were independent prognostic markers predicting the OS of AML patients (Figure7A, Supplementary Table S5). A nomogram was further depicted to predict the survival expectancy of AML patients by incorporating both the risk scores and clinicopathological characteristics (Figure 7B). We applied the C-Index to evaluate the predictive capability of the nomogram. The results showed that the nomogram harbored a high degree of discrimination 0.736*.* The calibration curve demonstrated that there was a good agreement between the estimated OS values of 1, 3, and 5 years and the actual observations of patients (Figure 7C). Moreover, DCA indicated that about 20–90% of patients could be benefited from the predictive model (Figure 7D).

## Discussion

Currently, the therapeutic approaches for AML remained limited and chemotherapy was still one of the most common first-line treatment modalities (Cai and Levine, 2019). Although an increasing number of targeted drugs emerged in recent years, including protein kinase inhibitors (Dinardo et al., 2018; Dogra et al., 2018; Perl et al., 2019), immune checkpoint antibodies (Vago and Gojo, 2020), and mitochondrial inhibitors (Ram et al., 2019), the percentage of responders was not satisfied. In this context, it is essential to clarify the molecular mechanisms underlying the AML progression and explore novel targets for AML treatment.

According to previous reports, m6A-related lncRNAs were associated with the development and deterioration of many human malignancies, including hepatocellular carcinoma (Li L. et al., 2021), bladder cancer (Li Z. et al., 2021), colon adenocarcinoma (Zhang P. et al., 2021), adrenocortical carcinoma (Jin et al., 2021), and breast cancer (Zhang X. et al., 2021). However, to our knowledge, few studies have focused on the role of m6A-related lncRNAs in AML. Hence, in the present study, we identified m6A-associated lncRNAs and provided a reliable platform for future studies that focused on the inner relationship between non-coding RNA and epigenetic modification. In detail, we first investigated the expression pattern of m6A genes in TCGA-AML and GEO cohorts. A strong co-expression correlation between distinct m6A regulators was detected. The METTL3–WTAP pair harbored the highest correlation coefficient. The expression level of WTAP turned to be increased when the expression of METTL3 decreased. Most m6A regulators were considered to be dysregulated and play an essential role in leukemia (Yao et al., 2021). Accumulating evidence indicated that m6A “writers,” METTL3, METTL14, and WTAP were all oncogenic in AML (Qing et al., 2021b). Melissa Sorci et al. reported that METTL3 levels were crucial for WTAP protein stabilization, but WTAP activation was not enough to trigger cell proliferation in the absence of a functional METTL3 in AML (Sorci et al., 2018). A plausible explanation was that although m6A writers could exert dual roles in the same cancer, m6A modification is not consistently pro-tumorigenic or antitumorigenic. The downstream effect was determined by the stability or translational efficiency of m6A motif-contained transcripts. These m6A motif contained oncogenes or tumor suppressors that could be influenced by differentially expressed m6A regulators would determine the biological processes of tumor cells. A similar phenomenon is observed in research involved with DNA methylation.

Then, we screened the deregulated lncRNAs from TCGA and GEO database. A lot of articles have reported that m6A-related lncRNAs were able to regulate cancer initiation and progression (Ni et al., 2019; Wen et al., 2020; Yang et al., 2020). Wen Ni et al. reported that m6A-modified transcripts of lncRNA GAS5 could regulate YAP activation in colorectal cancer progression (Ni et al., 2019). In another study, m6A modification in lncRNA NEAT1 played an oncogenic role in bone metastatic prostate cancer and was correlated with the poor prognosis (Wen et al., 2020). On the contrary, as an essential component of m6A writer, METTL14 has been shown to suppress proliferation and metastasis of colorectal cancer by downregulating oncogenic lncRNA XIST (Yang et al., 2020), suggesting the regulatory network of m6A and lncRNA in tumor pathophysiology was extremely complicated. Meanwhile, their relationships with m6A regulators were analyzed and a total of 41 m6A-related lncRNAs were identified in the end. Next, through univariate and multivariate Cox regression, we selected nine m6A-related lncRNAs that were associated with OS and further constructed an m6A-related lncRNA-based prognosis model based on their expression level. Among them, FAM30A, HCP5, LINC00963, TMEM147-AS1, and TTTY15 indicated a worse prognosis, but LINC00342, MEG3, HCG18, and N4BP2L2-IT2 demonstrated a better tumor prognosis. FAM30A was included in a prognostic model for AML, which was significantly correlated with the European Leukemia Net 2017 recommended prognostic genetic abnormalities (Guo et al., 2021). According to reports, HCP5 was overexpressed in AML and was positively associated with poor prognosis (Lei et al., 2020), it was significantly overexpressed in AML, and HCP5 upregulation promoted the progression of AML cells *via* the miR-1291/PIK3R5 axis (Liu Y. et al., 2021). Wenli Zuo et al. have shown that LINC00963 promoted AML development by regulating miR-608/MMP-15 (Zuo et al., 2020). Multiple lncRNAs have been reported to be both oncogenic and anti-oncogenic in AML. On one side, MEG3 was overexpressed in acute promyelocytic leukemia (APL), while exerting antitumor function in AML cell lines (Liu et al., 2019; Zimta et al., 2019). The aforementioned lncRNAs have been reported to be involved with AML, but TMEM147-AS1, TTTY15, LINC00342, HCG18, and N4BP2L2-IT2 have not been studied in AML yet.

Moreover, the pathophysiological role of 22 DEGs was investigated in the present study. AML tissues were grouped into high- and low-risk subgroups according to the median of risk scores. DEGs were mainly enriched in the viral protein interaction with cytokine and cytokine receptors, chemokine signaling pathway, transcriptional misregulation in cancers, and cytokine–cytokine receptor interaction pathways, which were shown by KEGG functional analysis. By GO analysis, multiple pathways involved with immune surveillance and defending were enriched, suggesting these genes may be associated with alteration in antitumor immunity. GSEA results revealed that myc targets, allograft rejection, apical junction, hypoxia, and PI3K/Akt/mTOR signaling pathway may be associated with the high-risk group. Among these signal pathways, the PI3K/Akt/mTOR signaling pathway has been reported to be aberrantly hyperactivated in multiple cancers such as AML, which is required to sustain the oncogenic potential of leukemia stem cell populations (Darici et al., 2020; Nepstad et al., 2020). The PI3K/Akt/mTOR signaling pathway was found to be potentially affected by m6A regulators in most cancers such as gastrointestinal cancer (Zhao Q. et al., 2020) and ovarian cancer (Bi et al., 2021), which was consistent with our results. M6A modification of lncRNAs may affect the occurrence and development of tumors, lncRNAs may also target m6A regulators as competitive endogenous RNAs, affecting tumor invasive progression (Xu F. et al., 2021; Zhao et al., 2021). Taken together, these findings implied potential correlation between m6A-modified lncRNAs and AML initiation or progression. It would be interesting to clarify the potential biological mechanism by which the abnormal expressions of m6A-related lncRNAs deteriorate AML.

In addition, we explored the association between the risk scores and the BMM of AML. Compared with the low-risk samples, AML in the high-risk group exhibited increased ImmuneScore and StromalScore. Another significant discovery was that certain immune cells, including naive B cells, eosinophils, activated mast cells, neutrophils, naive CD4 T cells, CD8 T cells, follicular helper T cells, and T-regulatory cells (Tregs), were enriched in high-risk groups. Infiltrated immune and stromal cells are vital components of the BM microenvironment and exert significant function in the progression of AML (Dufva et al., 2020). CD8-positive T cells were more abundant in bone marrow aspirates (BMAs) from patients with multiple relapses than in BMAs from those who had first relapsed or newly diagnosed AML (Williams et al., 2019). NFATC4 was an essential immune-related regulator in AML, and predicted worse survival by recruiting more Tregs (Zhao C. et al., 2020) and hence formed an immunosuppressive microenvironment, suggesting that Tregs were associated with a poor prognosis in AML patients. Interestingly, Yingxi Xu et al. revealed that Tregs promoted the stemness of leukemia cells *via* the IL10/IL10R/PI3K/AKT signaling pathway (Xu Y. et al., 2021). These findings were consistent with the results in the present study; however, other immune cells involved in immune imbalance remained unclear in AML. Therefore, elucidating the biological mechanisms based on the immune microenvironment may facilitate exploring novel therapeutic targets for AML.

The screening of gene signatures or biomarkers that could accurately predict the AML prognosis was still a huge challenge. We could identify the gene signals associated with the prognosis of AML *via* bioinformatics approaches. Thus, we evaluated the impact of m6A-related lncRNA risk score on the level of genetic variation, which consisted of SNP and CNV in AML patients. We found that patients in the high-risk group majorly harbored censoring of gene copy number. Conversely, patients in the low-risk group were majorly manifested as gene amplification. Na Li et al. illustrated that alterations in CNVs influenced the m6A-related gene expression in non-lung cancer genes (Li N. et al., 2021). A similar study found substantial prognostic values of the CNV of m6A regulatory genes in hepatocellular carcinoma (Liu G.-M. et al., 2021). However, according to the literature in PubMed, few studies have investigated the role of m6A-related lncRNA risk score on the level of CNV in AML. In the current study, AML was grouped into three prognostic risk groups: favorable, intermediate, and adverse. These clusters are according to both cytogenetics and relatively recent recognition of molecular disease subtypes that are distinct from the contribution of the cytogenetic risk (Pelcovits and Niroula, 2020). Our results may facilitate the invention of a new method for predicting the OS of AML patients.

Subsequently, we found that the high-risk group was featured with the higher IC50 values of multiple chemotherapeutics and small-molecule anticancer drugs, notably TW.37 and MG.132. It was reported that epigallocatechin-3-gallate (EGCG) increases ATRA-induced APL cell line (NB4) differentiation *via* phosphatase and tensin homolog (PTEN). The expression of PTEN was decreased in NB4 cells and could be rescued by proteases inhibitor MG132 (Yao et al., 2017). This indicated that MG132 may be a promising strategy for APL, which was a favorable group of AML. Our results suggested that the low-risk group may be more sensitive to MG132, which was consistent with previous results. These drugs may provide potential clues for precise treatment for AML patients with different risk scores. Finally, for the sake of better comprehension of the function of m6A-related lncRNA risk score in the survival of AML patients, we established a clinical prognostic model, which revealed good accuracy in predicting the survival expectancy of AML patients. Notably, the prognosis model constructed in the present study has the potential to be translated into clinical practices given its high predictive accuracy. Clinicians could refer to the risk score given by our model to assess the survival expectancy of AML patients and make appropriate clinical decisions.

Certainly, there were some limitations in the present study. First, the biological function of the m6A-related lncRNAs remained indistinct and warranted further exploration to determine approaches to reprogram the immune microenvironment and promote precision immunotherapy for AML. Second, this clinical prognostic model was only validated in TCGA and GSE37642, and more external validations based on RNA-seq cohorts are expected in the future to evaluate whether it can be applied to AML patients. Further experimental verifications are necessary to fully clarify the role of m6A-related lncRNAs and the potential mechanisms of AML. We would like to perform more careful examinations of the diagnosis and treatment effect of these predicted m6A-related lncRNAs in AML by combining *in vivo* and *in vitro* techniques.

In conclusion, the present study was the first systematic screening and comprehensive analysis of m6A-related lncRNAs in AML samples. We identified m6A-related lncRNAs with survival implications and constructed a novel and accurate prognostic model. The risk score was extremely associated with the malignant clinicopathological characteristics of AML and could be recognized as a novel biomarker. For the first time, the research of m6A-related lncRNAs has been published to exert essential roles in the immune infiltration and CNV of AML. The present study offered a potentially theoretical basis for further research demonstrating the role of m6A-related lncRNAs in AML, which provided new guidance for the valid treatment guidelines for AML.

## Data Availability

The datasets presented in this study can be found in online repositories. The names of the repository/repositories and accession number(s) can be found in the article.

## Glossary

AML: acute myeloid leukemia
APL: acute promyelocytic leukemia
AUC: area under the curve
BMAs: bone marrow aspirates
BMM: bone marrow microenvironment
BPs: biological processes
CCs: cellular components
C-index: consistency index
CNV: copy number variation
DCA: decision curve analysis
DDA1: DET1- and DDB1-associated protein 1
DEGs: differentially expressed genes
EGCG: pigallocatechin-3-gallate
FDR: false discovery rate
FPKM: fragments per kilobase of transcript per million mapped reads
GDSC: Genomics of Drug Sensitivity in Cancer
GEO: Gene Expression Omnibus
GO: gene ontology
GSEA: gene set enrichment analysis
KEGG: Kyoto Encyclopedia of Genes and Genomes
lncRNAs: long non-coding RNAs
m6A: N 6-methyladenosine
MFs: molecular functions
OS: overall survival
PTEN: phosphatase and tensin homolog
ROC: receiver operating characteristic
SNP: single-nucleotide polymorphism
TCGA: The Cancer Genome Atlas
TMB: tumor mutation burden
TME: tumor microenvironment
TPM: transcripts per million reads
Tregs: T-regulatory cells
