# Supplementary material for: N6-Methyladenosine-Related lncRNAs Are Novel Prognostic Markers and Predict the Immune Landscape in Acute Myeloid Leukemia
Source: Front Genet. 2022 May 9;13:804614. doi: 10.3389/fgene.2022.804614 (PMC9125310; doi:10.3389/fgene.2022.804614)
Supplement: Supplementary file 1 [file DataSheet1.docx]

Supplementary Material

**Table1 The clinicopathologic features in AML patients**

| Characteristic | low | high | p |
| --- | --- | --- | --- |
| n | 100 | 30 |  |
| Gender, n (%) |  |  | 0.162 |
| Female | 50 (38.5%) | 10 (7.7%) |  |
| Male | 50 (38.5%) | 20 (15.4%) |  |
| Morphology_code, n (%) |  |  | 0.033 |
| M0 | 8 (6.2%) | 4 (3.1%) |  |
| M1 | 24 (18.5%) | 6 (4.6%) |  |
| M2 | 25 (19.2%) | 7 (5.4%) |  |
| M3 | 14 (10.8%) | 0 (0%) |  |
| M4 | 20 (15.4%) | 7 (5.4%) |  |
| M5 | 9 (6.9%) | 3 (2.3%) |  |
| M6 | 0 (0%) | 2 (1.5%) |  |
| M7 | 0 (0%) | 1 (0.8%) |  |
| RUNX1 Mutation, n (%) |  |  | 0.082 |
| Mut | 4 (3.1%) | 4 (3.1%) |  |
| WT | 96 (73.8%) | 26 (20%) |  |
| Age, meidan (IQR) | 53 (41, 64) | 62 (45, 70.75) | 0.074 |

**Table2 GO analysis**

| **class** | **ID** | **Description** | **numbers** | **Pvalue** |
| --- | --- | --- | --- | --- |
| Biological Process | GO:0098542 | defense response to other organism | 11 | 1.87E-11 |
| Biological Process | GO:0051707 | response to other organism | 12 | 3.17E-10 |
| Biological Process | GO:0043207 | response to external biotic stimulus | 12 | 3.21E-10 |
| Biological Process | GO:0009607 | response to biotic stimulus | 12 | 4.93E-10 |
| Biological Process | GO:0019730 | antimicrobial humoral response | 6 | 1.26E-08 |
| Biological Process | GO:0006952 | defense response | 13 | 2.70E-08 |
| Biological Process | GO:0051852 | disruption by host of symbiont cells | 4 | 3.38E-08 |
| Biological Process | GO:0051873 | killing by host of symbiont cells | 4 | 3.38E-08 |
| Biological Process | GO:0009617 | response to bacterium | 9 | 4.44E-08 |
| Biological Process | GO:0051818 | disruption of cells of other organism involved in symbiotic interaction | 4 | 4.47E-08 |
| Biological Process | GO:0051883 | killing of cells in other organism involved in symbiotic interaction | 4 | 4.47E-08 |
| Biological Process | GO:0050829 | defense response to Gram-negative bacterium | 5 | 5.11E-08 |
| Biological Process | GO:0042742 | defense response to bacterium | 7 | 8.74E-08 |
| Biological Process | GO:0070944 | neutrophil mediated killing of bacterium | 3 | 1.19E-07 |
| Biological Process | GO:0009605 | response to external stimulus | 14 | 1.42E-07 |
| Biological Process | GO:0070943 | neutrophil mediated killing of symbiont cell | 3 | 1.71E-07 |
| Biological Process | GO:0006959 | humoral immune response | 7 | 1.82E-07 |
| Biological Process | GO:0051704 | multi-organism process | 14 | 2.66E-07 |
| Biological Process | GO:0070942 | neutrophil mediated cytotoxicity | 3 | 3.12E-07 |
| Biological Process | GO:0033993 | response to lipid | 9 | 5.66E-07 |
| Cellular Component | GO:0034774 | secretory granule lumen | 7 | 5.75E-08 |
| Cellular Component | GO:0035578 | azurophil granule lumen | 5 | 6.22E-08 |
| Cellular Component | GO:0060205 | cytoplasmic vesicle lumen | 7 | 8.53E-08 |
| Cellular Component | GO:0031983 | vesicle lumen | 7 | 8.70E-08 |
| Cellular Component | GO:0005766 | primary lysosome | 5 | 8.62E-07 |
| Cellular Component | GO:0042582 | azurophil granule | 5 | 8.62E-07 |
| Cellular Component | GO:0005775 | vacuolar lumen | 5 | 1.57E-06 |
| Cellular Component | GO:0030141 | secretory granule | 8 | 3.56E-06 |
| Cellular Component | GO:0099503 | secretory vesicle | 8 | 1.35E-05 |
| Cellular Component | GO:0031410 | cytoplasmic vesicle | 11 | 3.33E-05 |
| Cellular Component | GO:0097708 | intracellular vesicle | 11 | 3.40E-05 |
| Cellular Component | GO:0044433 | cytoplasmic vesicle part | 8 | 2.53E-04 |
| Cellular Component | GO:0005773 | vacuole | 6 | 2.91E-04 |
| Cellular Component | GO:0044437 | vacuolar part | 5 | 5.94E-04 |
| Cellular Component | GO:0000323 | lytic vacuole | 5 | 1.33E-03 |
| Cellular Component | GO:0005764 | lysosome | 5 | 1.33E-03 |
| Cellular Component | GO:0020003 | symbiont-containing vacuole | 1 | 2.37E-03 |
| Cellular Component | GO:0020005 | symbiont-containing vacuole membrane | 1 | 2.37E-03 |
| Cellular Component | GO:0030430 | host cell cytoplasm | 1 | 2.37E-03 |
| Cellular Component | GO:0033646 | host intracellular part | 1 | 2.37E-03 |
| Molecular Function | GO:0008201 | heparin binding | 5 | 1.39E-06 |
| Molecular Function | GO:0005539 | glycosaminoglycan binding | 5 | 7.62E-06 |
| Molecular Function | GO:1901681 | sulfur compound binding | 5 | 1.26E-05 |
| Molecular Function | GO:0045236 | CXCR chemokine receptor binding | 2 | 2.06E-04 |
| Molecular Function | GO:0030674 | protein binding, bridging | 3 | 9.11E-04 |
| Molecular Function | GO:0004252 | serine-type endopeptidase activity | 3 | 9.95E-04 |
| Molecular Function | GO:0003912 | DNA nucleotidylexotransferase activity | 1 | 1.20E-03 |
| Molecular Function | GO:0004397 | histidine ammonia-lyase activity | 1 | 1.20E-03 |
| Molecular Function | GO:0008269 | JAK pathway signal transduction adaptor activity | 1 | 1.20E-03 |
| Molecular Function | GO:0008236 | serine-type peptidase activity | 3 | 1.34E-03 |
| Molecular Function | GO:0017171 | serine hydrolase activity | 3 | 1.42E-03 |
| Molecular Function | GO:0035591 | signaling adaptor activity | 2 | 1.43E-03 |
| Molecular Function | GO:0008009 | chemokine activity | 2 | 1.55E-03 |
| Molecular Function | GO:0042379 | chemokine receptor binding | 2 | 3.31E-03 |
| Molecular Function | GO:0097367 | carbohydrate derivative binding | 8 | 3.50E-03 |
| Molecular Function | GO:0060090 | molecular adaptor activity | 3 | 3.63E-03 |
| Molecular Function | GO:0005126 | cytokine receptor binding | 3 | 4.07E-03 |
| Molecular Function | GO:0001093 | TFIIB-class transcription factor binding | 1 | 4.77E-03 |
| Molecular Function | GO:0016841 | ammonia-lyase activity | 1 | 5.96E-03 |
| Molecular Function | GO:0048248 | CXCR3 chemokine receptor binding | 1 | 5.96E-03 |

**Table3 KEGG analysis**

| class | ID | Description | Pvalue | Number |
| --- | --- | --- | --- | --- |
| Environmental Information Processing | ko04061 | Viral protein interaction with cytokine and cytokine receptor | 1.21E-02 | 2 |
| Genetic Information Processing | ko03450 | Non-homologous end-joining | 2.33E-02 | 1 |
| Metabolism | ko00340 | Histidine metabolism | 3.97E-02 | 1 |
| Organismal Systems | ko04614 | Renin-angiotensin system | 3.97E-02 | 1 |
| Organismal Systems | ko04062 | Chemokine signaling pathway | 4.22E-02 | 2 |
| Human Diseases | ko05322 | Systemic lupus erythematosus | 5.02E-02 | 2 |
| Human Diseases | ko05202 | Transcriptional misregulation in cancers | 7.30E-02 | 2 |
| Human Diseases | ko04930 | Type II diabetes mellitus | 8.11E-02 | 1 |
| Organismal Systems | ko04979 | Cholesterol metabolism | 8.73E-02 | 1 |
| Organismal Systems | ko04913 | Ovarian Steroidogenesis | 8.88E-02 | 1 |
| Environmental Information Processing | ko04060 | Cytokine-cytokine receptor interaction | 8.98E-02 | 2 |
| Organismal Systems | ko04927 | Cortisol synthesis and secretion | 1.06E-01 | 1 |
| Cellular Processes | ko04137 | Mitophagy - animal | 1.09E-01 | 1 |
| Human Diseases | ko05221 | Acute myeloid leukemia | 1.15E-01 | 1 |
| Organismal Systems | ko04917 | Prolactin signaling pathway | 1.24E-01 | 1 |
| Metabolism | ko00983 | Drug metabolism - other enzymes | 1.27E-01 | 1 |
| Organismal Systems | ko04925 | Aldosterone synthesis and secretion | 1.55E-01 | 1 |
| Human Diseases | ko05310 | Asthma | 1.63E-01 | 1 |
| Human Diseases | ko05340 | Primary immunodeficiency | 1.79E-01 | 1 |
| Organismal Systems | ko04935 | Growth hormone synthesis, secretion and action | 1.90E-01 | 1 |

**Table4 Gene set enrichment analysis**

| Name | Size | Enrichment  Score | NES | P value | Leading  edge |
| --- | --- | --- | --- | --- | --- |
| HALLMARK_MYC_TARGETS_V1 | 196 | 0.771282 | 1.306494 | 2.41E-05 | tags=65%, list=16%, signal=55% |
| HALLMARK_ALLOGRAFT_REJECTION | 195 | 0.761167 | 1.288934 | 3.94E-05 | tags=29%, list=12%, signal=26% |
| HALLMARK_APICAL_JUNCTION | 198 | 0.765542 | 1.296786 | 5.87E-05 | tags=21%, list=10%, signal=19% |
| HALLMARK_HYPOXIA | 196 | 0.715757 | 1.21244 | 0.004018 | tags=28%, list=14%, signal=24% |
| HALLMARK_PI3K_AKT_MTOR_SIGNALING | 105 | 0.72356 | 1.214199 | 0.012151 | tags=45%, list=15%, signal=38% |
| HALLMARK_EPITHELIAL_MESENCHYMAL_TRANSITION | 198 | 0.699134 | 1.184294 | 0.012453 | tags=14%, list=13%, signal=12% |
| HALLMARK_MITOTIC_SPINDLE | 198 | 0.69503 | 1.177342 | 0.014566 | tags=56%, list=21%, signal=45% |
| HALLMARK_REACTIVE_OXYGEN_SPECIES_PATHWAY | 49 | 0.766478 | 1.261514 | 0.016983 | tags=29%, list=10%, signal=26% |
| HALLMARK_HEDGEHOG_SIGNALING | 36 | 0.805236 | 1.307111 | 0.018355 | tags=28%, list=15%, signal=24% |

**Table5 Univariate and multivariate Cox analysis in patients with AML** **based on the m6A-related lncRNAs risk model**

| Characteristics | Total(N) | HR(95% CI) Univariate analysis | P value Univariate analysis | HR(95% CI) Multivariate analysis | P value Multivariate analysis |
| --- | --- | --- | --- | --- | --- |
| Age | 130 | 2.03 (2.02-2.05) | <0.001 | 3.46 (2.06-5.8) | <0.001 |
| Gender | 130 |  | 0.438 |  |  |
| Male | 70 | Reference |  |  |  |
| Female | 60 | 0.83 (0.52-1.3) | 0.438 |  |  |
| morphology_code | 130 |  | 0.105 |  |  |
| M0 | 12 | Reference |  |  |  |
| M1 | 30 | 2.34 (0.94-5.9) | 0.069 |  |  |
| M2 | 32 | 1.72 (0.67-4.4) | 0.256 |  |  |
| M3 | 14 | 0.63 (0.15-2.6) | 0.521 |  |  |
| M4 | 27 | 2.01 (0.80-5.0) | 0.137 |  |  |
| M5 | 12 | 2.27 (2.07-3.07) | 0.064 | 3.11 (1.03-9.3) | 0.044 |
| RUNX1 Mutation | 130 |  | 0.169 |  |  |
| WT | 122 | Reference |  |  |  |
| Mut | 8 | 1.34 (0.54-3.3) | 0.169 |  |  |
| risk | 130 |  | <0.001 |  |  |
| low | 100 | Reference |  |  |  |
| high | 30 | 3.768 (2.35-6.04) | <0.001 | 3.83 (2.29-6.5) | <0.001 |


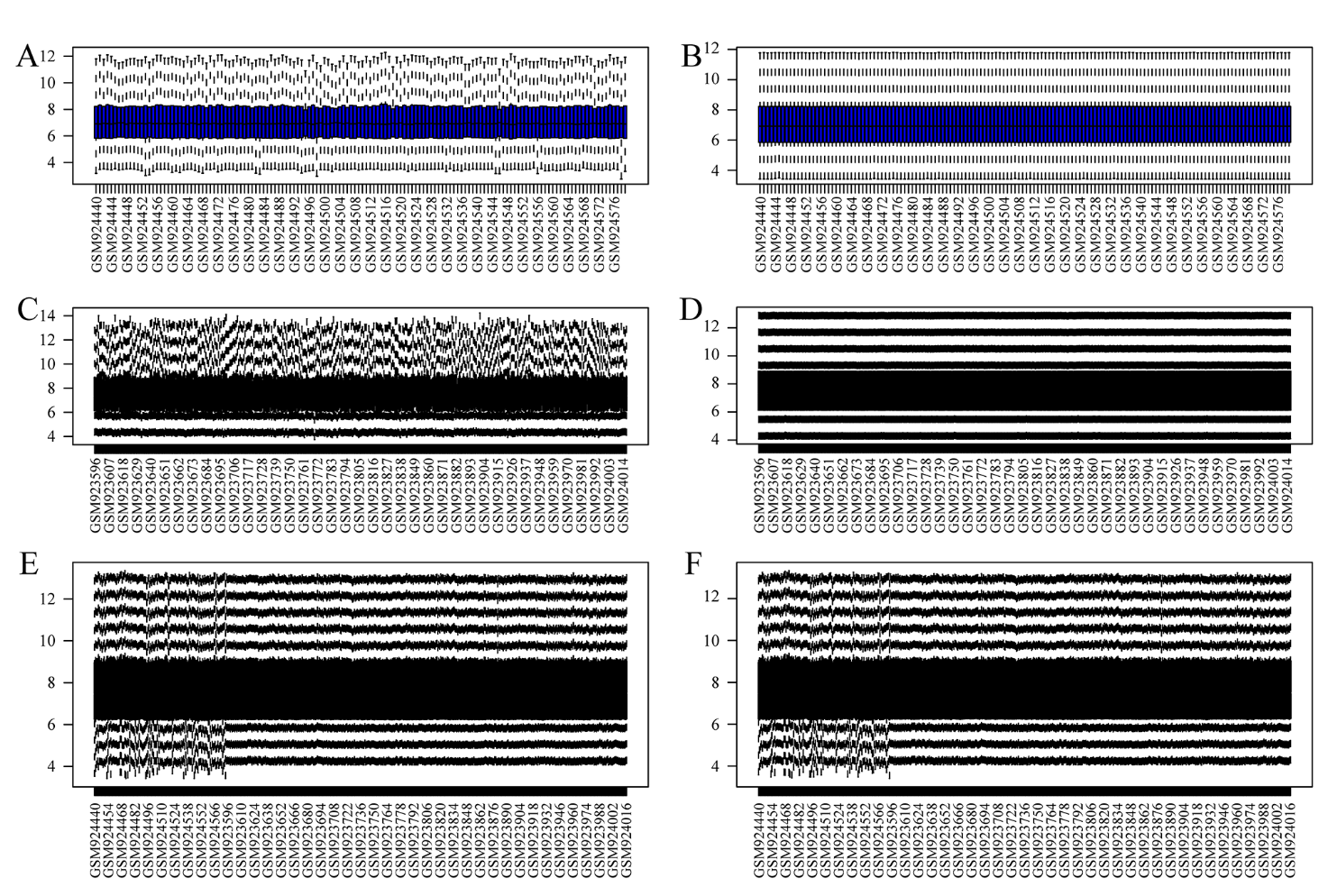


**FIGURE S1 |** Box plots showed the elimination of batch effect before and after the GSE37642 dataset were merged. Box plots before **(A)** and after **(B)** the standardization of the GSE37642 dataset based on GPL570 platform. Box figure before **(C)** and after **(D)** the standardization of the GSE37642 dataset based on GPL96 platform. Box plots of the GSE37642 dataset before **(E)** and after **(F)** the data from two platforms were merged.


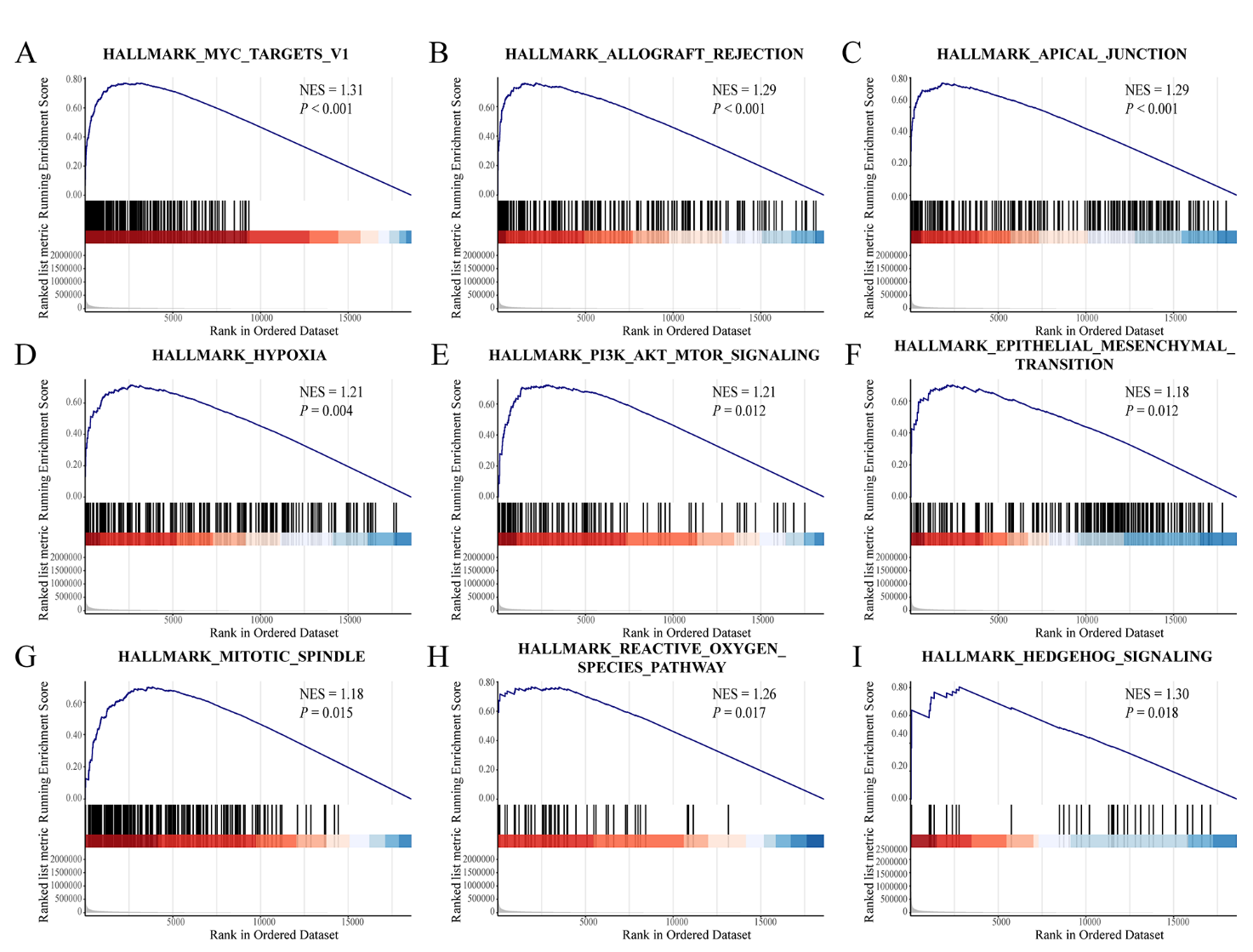


**FIGURE S2 |** GSEA analysis based on DEGs in TCGA-AML dataset. **(A-I)** GSEA analysis showed that increased risk-scores were closely related to signaling pathways including myc targets, allograft rejection, apical junction, hypoxia and PI3K/Akt/ mTOR signaling pathway.
